# Molecular mechanism analysis of *ZmRL6* positively regulating drought stress tolerance in maize

**DOI:** 10.1007/s44154-023-00125-x

**Published:** 2023-11-16

**Authors:** Pengyu Zhang, Tongchao Wang, Liru Cao, Zhixin Jiao, Lixia Ku, Dandan Dou, Zhixue Liu, Jiaxu Fu, Xiaowen Xie, Yingfang Zhu, Leelyn Chong, Li Wei

**Affiliations:** 1 National Key Laboratory of Wheat and Maize Crop Science, Henan Agricultural University, Zhengzhou, 450046 China; 2 Henan Academy of Agricultural Sciences, Zhengzhou, 450002 China; 3 State Key Laboratory of Crop Stress Adaptation and Improvement, School of Life Sciences, Henan University, Kaifeng, 475001 China

**Keywords:** *ZmRL6*, 1R-MYB, MYB-related genes, Drought stress, Maize

## Abstract

MYB-related genes, a subclass of MYB transcription factor family, have been documented to play important roles in biological processes such as secondary metabolism and stress responses that affect plant growth and development. However, the regulatory roles of MYB-related genes in drought stress response remain unclear in maize. In this study, we discovered that a 1R-MYB gene, *ZmRL6*, encodes a 96-amino acid protein and is highly drought-inducible. We also found that it is conserved in both barley (*Hordeum vulgare *L*.*) and *Aegilops tauschii*. Furthermore, we observed that overexpression of *ZmRL6* can enhance drought tolerance while knock-out of *ZmRL6* by CRISPR-Cas9 results in drought hypersensitivity. DAP-seq analyses additionally revealed the *ZmRL6* target genes mainly contain ACCGTT, TTACCAAAC and AGCCCGAG motifs in their promoters. By combining RNA-seq and DAP-seq results together, we subsequently identified eight novel target genes of *ZmRL6* that are involved in maize's hormone signal transduction, sugar metabolism, lignin synthesis, and redox signaling/oxidative stress. Collectively, our data provided insights into the roles of *ZmRL6* in maize’s drought response.

**Supplementary Information:**

The online version contains supplementary material available at 10.1007/s44154-023-00125-x.

## Introduction

Maize (*Zea mays* L.), recognized as one of the most essential crops in the world, serves as a primary source of food, feed and industrial raw materials for the increasing population. A number of biotic and abiotic stress factors such as drought has been known to significantly impact the maize’s growth and development (Sun et al. 2022). It has been reported that a 40% moisture reduction in the soil could decrease maize’s grain yield up to 39% (Daryanto et al. 2016). Isolating traits that are specific for drought tolerance in maize has been challenging. Further studies are needed to better understand this complex mechanism so that crucial regulators for drought tolerance in maize can be identified.

The transcription factors (TFs) of bZIP, WRKY, ERF, NAC and MYB family members were suggested to play roles in environmental stress response(s) as they were found to activate various stress-responsive signaling pathways (Kobayashi et al. 2008). In fact, one of our published works has identified a highly drought-responsive TF that belongs to the MYB family (Cao et al. 2019, 2020; Wang et al. 2020). MYB proteins are extensively distributed in higher plants and characterized by a highly conservative MYB DNA-binding domain that usually has one to four repeats (R). MYB Rs contain roughly 52-amino acid residues and they are considered as imperfect (Baldoni et al. 2015). Based on the position and number of adjacent Rs in the MYB domains, MYB TFs can be classified into four subgroups: 1R-MYB (MYB-related), R2R3-MYB, 3R-MYB (R1R2R3-MYB) and 4R-MYB with 4 Rs (Zhang et al. 2021), and most *MYB* genes in plants are known for encoding R2R3-MYB class proteins (Dubos et al., 2022). Although many MYB proteins have been identified in maize, only a few of the MYB TFs have thus far been reported to possess apparent function (Du et al. 2012; Chen et al. 2018). The first MYB TF cloned in higher plants was *ZmMYBC1* and it was reported to play an important regulatory role in the maize’s biosynthesis of anthocyanin (Paz-Ares et al. 1987). TFs from both subclasses of 1R-MYB and R2R3-MYB have been demonstrated to participate in drought stress response and more R2R3-MYB members were reported for this role than the 1R-MYB members.

We have previously conducted a transcriptome analysis that revealed a number of drought-responsive TFs in the inbred line Yu882 (Cao et al. 2019; Wang et al. 2020). One of the most drought-responsive TF genes identified was *GRMZM2G114503*. We have also discovered that this gene is homologous to the 1R-MYB subgroup gene of *AtRL6* in Arabidopsis, consequently, we named it as *ZmRL6*. In the present study, we elucidated its function in the drought tolerance of maize found that the overexpression of *ZmRL6* gene significantly improved maize’s drought tolerance. Compared to the overexpression lines, *ZmRL6*-knockout mutants (generated using CRISPR/Cas9 system) exhibited drought sensitive phenotypes such as severe leaf wilting, curled leaves, and greater yellow-colored leaf tips, indicating the positive role of *ZmRL6* in drought stress regulation. The combined analyses of RNA-seq and DNA affinity purification sequencing (DAP-seq) data additionally revealed several target genes of *ZmRL6*. These target genes were found to involve in the redox signaling, lignin synthesis, sugar metabolism and hormone signaling pathways of maize. Our study indicated that ZmRL6 was a potential drought stress regulator in maize.

## Results

### The involvement of ZmRL6 in drought stress response

**Fig. 1:**
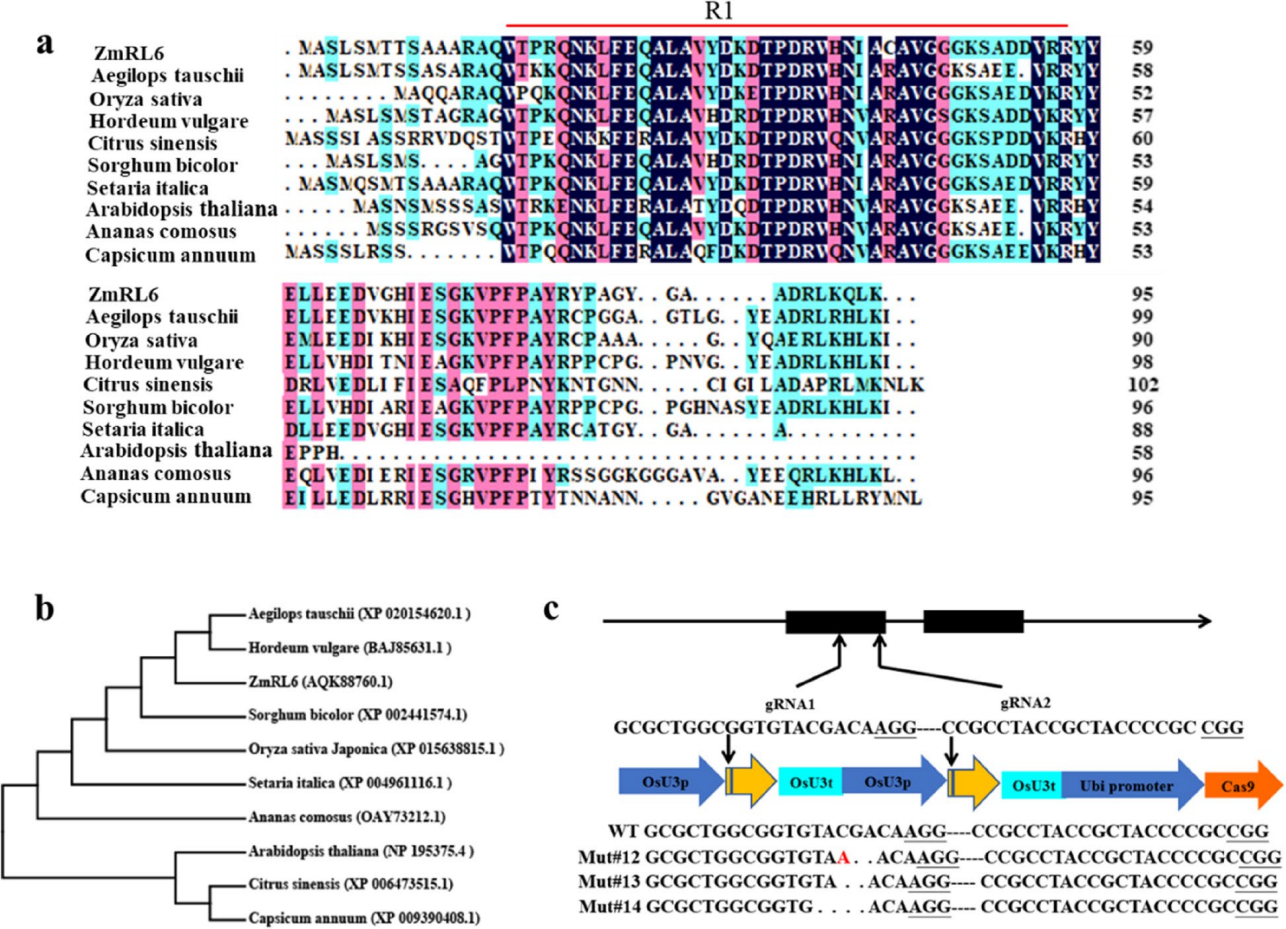
Sequence comparison of the 1R-MYB TFs and *ZmRL6* along with the generation of *ZmRL6* over-expression and knock-out transgenic lines. **a** Amino acid sequence alignment of *ZmRL6* and other 1R-MYB proteins (**b**) Phylogenetic analysis of *ZmRL6*: phylogenetic tree constructed using the N-J method from the deduced amino acid sequence of ZmRL6 and other 1R-MYB protein sequences with the use of MEGA4.0. Bootstrap values (above 50%) from 1,000 replicates are indicated at each node (**b**) The mutation types created by CRISPR-Cas9

From our previous transcriptome sequencing of the maize leaves subjected to osmotic stress and rewatering conditions (Cao et al. 2019; Wang et al., 2020), we successfully identified a total of 11,002 differentially expressed genes (DEGs) and 556 of them were TFs (Zhang et al. 2020). One of the TFs (*GRMZM2G114503*) was noted to be highly drought-responsive (Fig. S1). Moreover, it was identified as a hub of drought responsive genes based on co-expression analysis using Cytoscape software. We further explored this gene and found that it belongs to the MYB TF family. This gene was revealed to be homologous to *AtRL6* in Arabidopsis, and thus we named it as *ZmRL6*. The ORF of *ZmRL6* was analyzed to be 291 nucleotides long and it encodes a polypeptide of 96 amino acids with a predicted molecular mass of 10.7 kDa and an isoelectric point of 8.14. Sequence analysis additionally revealed *ZmRL6* gene belongs to the 1R-MYB subgroup as it contains a sequence of only one typical R in the MYB domain, which shares similarity with other known members of the 1R-MYB protein family (Fig. 1a). The phylogenetic tree analysis further suggested that *ZmRL6* is closely related to barley (*Hordeum vulgare* L.) and *Aegilops tauschii* (Fig. 1b).

To examine the function of *ZmRL6*, we obtained 20 over-expression lines (*ZmRL6*-OE) and 20 knockout mutant transgenic lines (*ZmRL6*-Mut) of maize through performing genetic transformation. The design of two SgRNAs targeting the genomic sequence of *ZmRL6* as well as the mutation types created by CRISPR-Cas9 are presented in Fig. 1c. The independent lines of *ZmRL6*-OE #2, *ZmRL6*-OE #10 and *ZmRL6*-OE #18 and *ZmRL6*-Mut #12, *ZmRL6*-Mut #13 and *ZmRL6*-Mut #14 were subsequently utilized for phenotypic and transcriptome analyses.

**Fig. 2:**
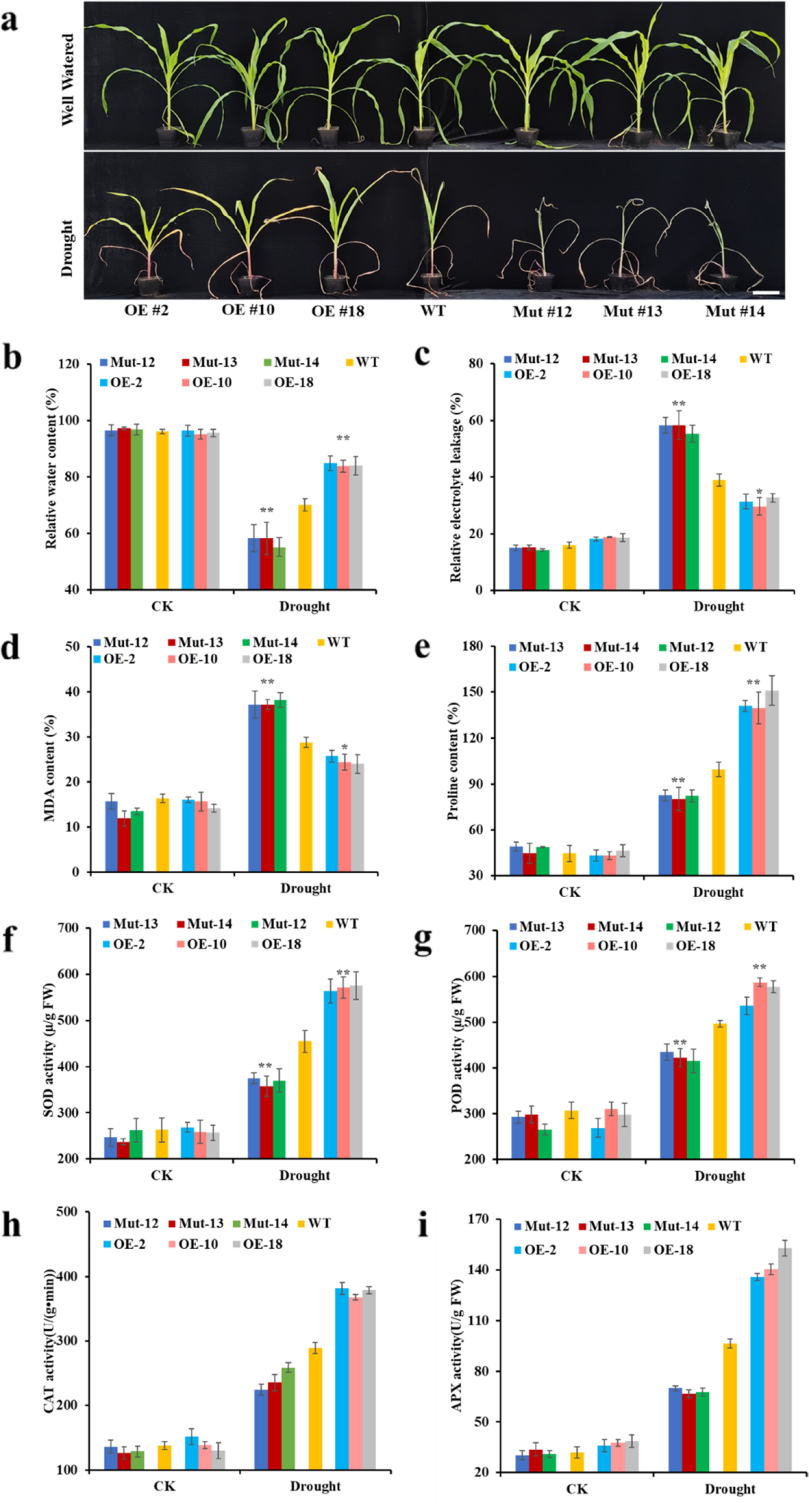
*ZmRL6* gene plays a positive role in drought stress response. **a** Representative phenotype of WT and ZmRL6 transgenic lines under normal and drought stress. The drought treatment experiments were repeated three times with similar results (**b**) Quantification of the relative water content (**c**) relative electrical leakage (**d**) malondialdehyde (MDA) content (**e**) proline content (**f**) the activities of antioxidant enzymes SOD (**g**) peroxidase POD (**h**) CAT activity (**i**) APX activity of maize leaves under drought stress. (*:* p* ≤ 0.05; **: *p* ≤ 0.01). Data represent mean ± SD (*n* = 6). * *p* < 0.05, ** *p* < 0.01, Student’s *t* test relative to WT

To probe the role of *ZmRL6* in drought response, we grew the WT and *ZmRL6* transgenic maize lines to a three-leaf stage before separating them into two groups. One of the seedling groups was under normal irrigation while the other group’s soil water content was controlled at about 20% for mimicking drought. No obvious difference was noted between the WT, *ZmRL6* OE lines and knockout mutants in normal irrigation as they had similar growth (Fig. 2a). However, *ZmRL6* OE seedlings showed mild leaf wilting symptoms after drought treatment compared to the WT and *ZmRL6* mutant plants exhibited more severe signs of leaf wilting, curled leaves, and yellow leaf tips, and the survival rate of *ZmRL6* CRISPR lines (Mut#12) was lower than WT, while OE lines (OE#2) showed higher survival rate (Fig. S2); indicating a positive role of *ZmRL6* in drought response. Moreover, the relative water content (RWC), electrolyte leakage (EL), contents of malondialdehyde (MDA) and proline, as well as antioxidant enzyme activity were measured to assess maize’s drought response (Fig. 2b-g). No significant difference was found for these measurements in the WT, *ZmRL6*-OE and *ZmRL6*-Mut lines under normal irrigation. After drought treatment, the RWC of maize leaves decreased in all of the WT and transgenic lines, and the value of the RWC in *ZmRL6* OE lines was significantly higher than those in the WT and *ZmRL6* mutant lines. Additionally, the RWC of *ZmRL6* mutant lines was much lower than the WT (Fig. 2b). The EL and MDA content also increased after drought treatment in all maize seedlings compared to those without treatment. In *ZmRL6*-OE plants, their EL and MDA contents were found to be lower than those in the WT while the values for those measurements were higher in the *ZmRL6* mutants compared to the WT (Fig. 2c-d). Similarly, proline content, superoxide dismutase (SOD) and peroxidase (POD) enzymatic activities increased after drought stress in all of the WT and *ZmRL6* transgenic lines. The values of those traits were higher in the *ZmRL6*-OE plants than those in the WT; while the value of proline content, SOD and POD enzymatic activities in *ZmRL6*-Mut plants were lower than the WT (Fig. 2e-g). The activity of ascorbate peroxidase (APX) and catalase (CAT) exhibited the similar trend with the SOD and POD enzymes, respectively (Fig. 2h-i). The rate of water loss from detached leaves was much higher for *ZmRL6* mutant lines, while the water loss was much lower for *ZmRL6* OE line compared to the WT (Fig. S3). In addition, *ZmRL6*-OE and *ZmRL6*-Mut lines had normal growth phenotype (Fig. S4). These results suggested that *ZmRL6* may play a positive role in maize’s drought stress response without affecting its development.

### Identification of ZmRL6-regulated genes in response to drought by RNA-seq

To understand the regulatory network of *ZmRL6* in response to drought, RNA-seq analysis was conducted using total RNA extracted from the leaves of *ZmRL6*-OE10, *ZmRL6*-Mut14 and the WT after drought treatment. A clean data ratio for all samples was achieved at more than 98% (Table S2). The rates of reads mapping to the reference genome were more than 87% (Table S3), indicating our sequencing results were reliable and appropriate for follow-up experimental analyses.

**Fig. 3:**
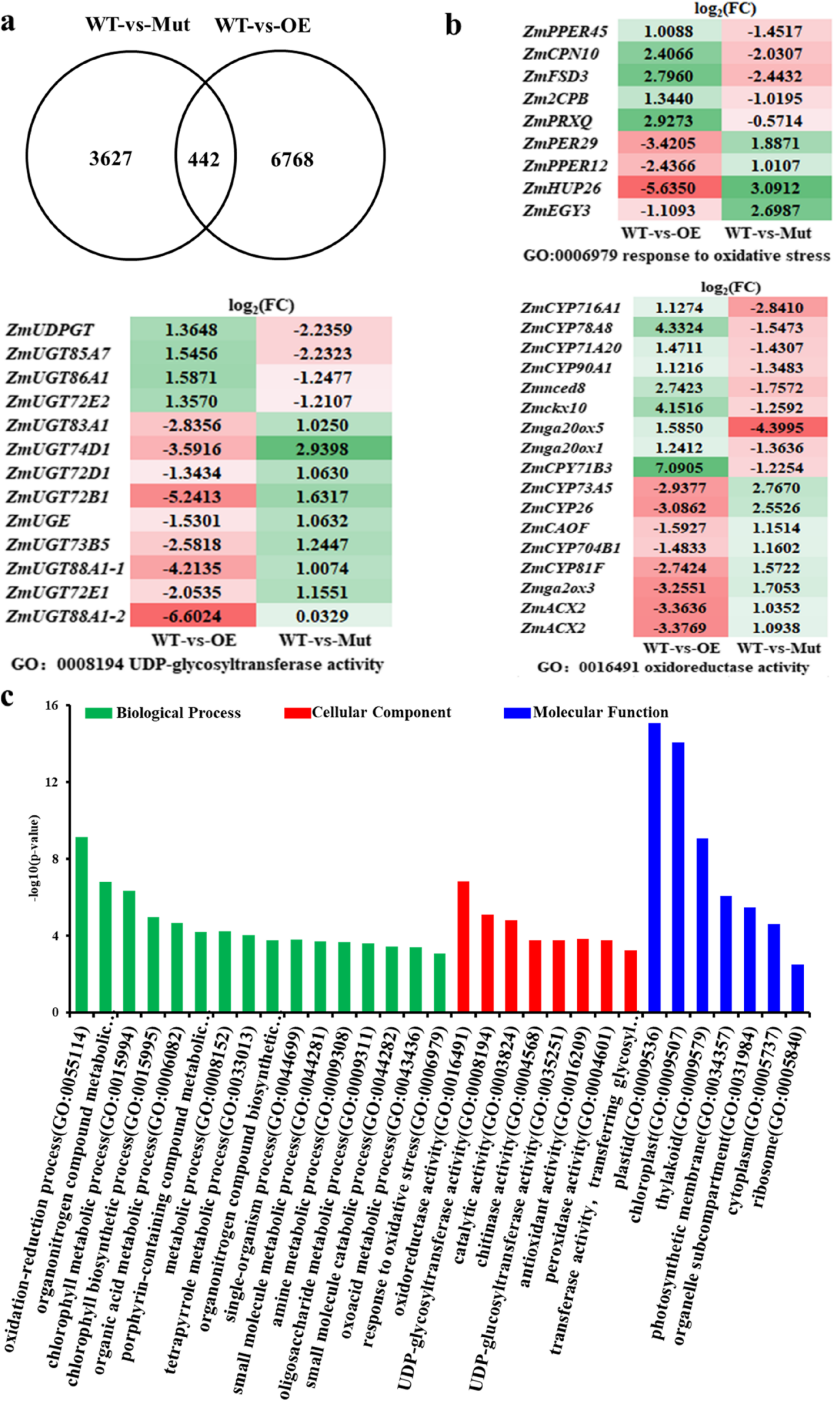
RNA-seq analysis of differentially expressed (DE) genes regulated by *ZmRL6*. **a** Venn diagram showing the overlapping genes between the groups of WT-vs-Mut and WT-vs-OE (**b**) Heat map of the DEGs involved in oxidoreductase activity, sugar metabolism and response to oxidative stress (**c**) GO enrichment of the overlapping DE genes between WT-vs-Mut and WT-vs-OE

We found that a total of 4069 and 7210 genes were differentially expressed with significance in the groups of WT-vs-Mut and WT-vs-OE, respectively. Venn diagrams showed that there were 442 differentially expressed genes (DEGs) overlapping with each other. Among them, we obtained 207 up-regulated DEGs which were up-regulated in the WT-vs-OE treatment group and down-regulated in the WT-vs-Mut treatment group, and 235 down-regulated DEGs which were down-regulated in the WT-vs-OE treatment group and up-regulated in the WT-vs-Mut treatment group (Fig. 3a).

GO enrichment analysis of the 442 DEGs revealed they are mostly involved in the following three biological processes: oxidation–reduction, organonitrogen compound metabolic process and chlorophyll metabolic process. They are also likely to serve in the following functional roles: oxidoreductase, UDP-glycosyltransferase and catalytic (Fig. 3c). Heat map of the DEGs further suggested that they are involved in the biochemical processes of oxidative stress response, oxidoreductase activity and UDP-glycosyltransferase activity. Furthermore, the expression of these genes was shown to be opposite of those in the *ZmRL6*-OE and *ZmRL6*-Mut lines (Fig. 3b).

### ZmRL6 protein is located in the nucleus and possesses transcriptional activity

**Fig. 4:**
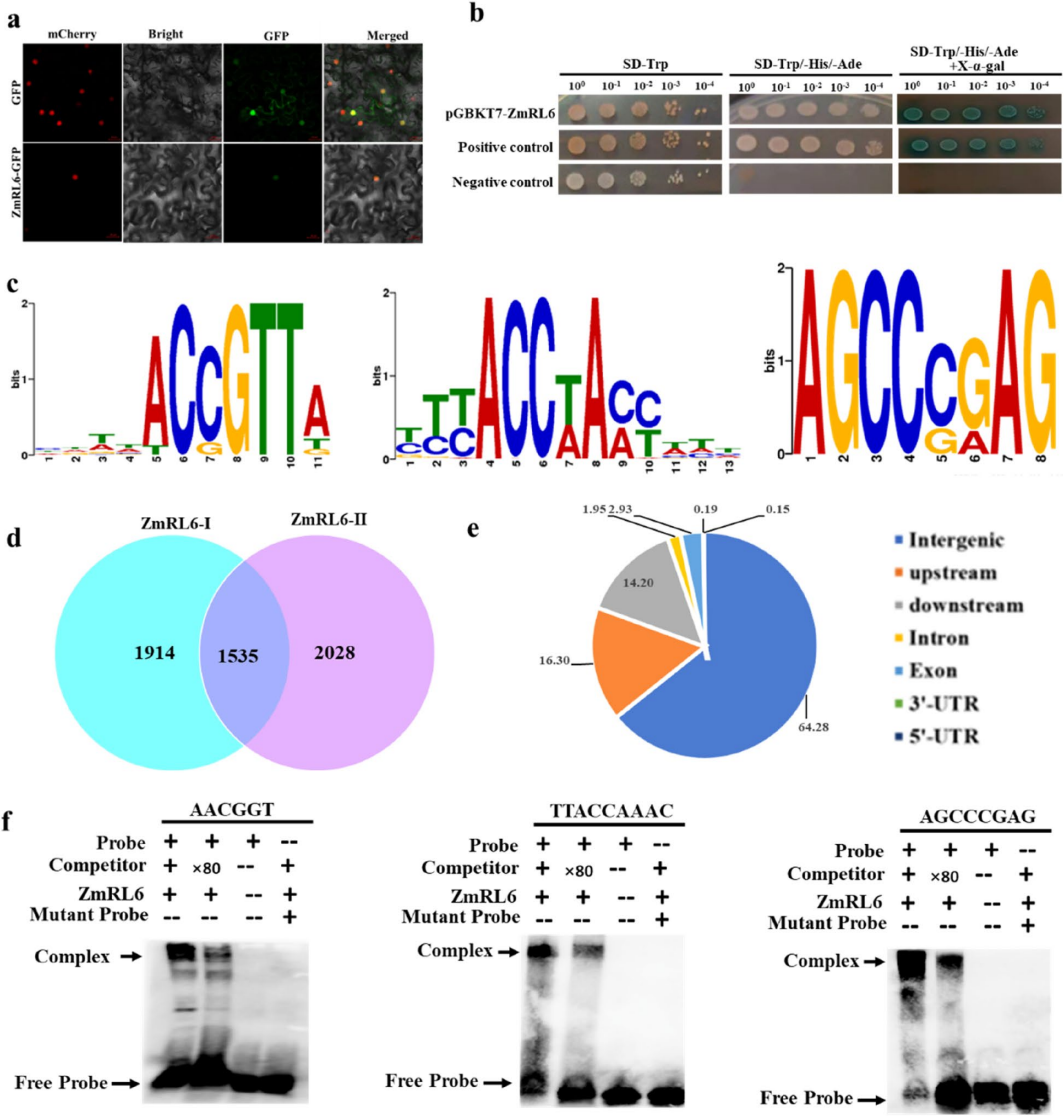
Analysis of *ZmRL6* target genes by DAP-seq and EMSA. **a** The subcellular localization of ZmRL6. 35S::GFP and 35S::ZmRL6-GFP plasmids were transformed into the Nicotiana benthamiana leaf epidermal cells before visualizing the GFP fluorescence by confocal microscope. From left to right, GFP fluorescence, bright-field, and overlay of the GFP fluorescence and bright-field. mCherry serves as a positive nuclear marker. Bar = 20 μ m (**b**) Transcriptional activity analysis of the ZmRL6 protein in Y2H assays. pGBKT7-ZmRL6 only, pGBKT7-53 /pGADT7-T (positive control) and pGBKT7-lam/pGADT7 (negative control) were transformed into Saccharomyces cerevisiae for determining its transcriptional activity (**c**) ZmRL6 binding to the ACCGTT, TTACCAAAC and AGCCCGAG core motifs as identified by MEME-ChIP (**d**) Comparison of ZmRL6-occupied peaks using two biological repetitions (**e**) Distribution of the ZmRL6 binding sites (**f**) EMSA results confirming the in vitro binding of ZmRL6 to ACCGTT, TTACCAAAC and AGCCCGAG probes. The arrow indicates the position of a protein-DNA complex after the incubation of biotin-labelled DNA probe and the ZmRL6 protein

The subcellular localization of ZmRL6-GFP was determined by green fluorescence intensity. In contrast to the control construct, which exhibited fluorescence throughout the whole nucleus and plasma membrane, the fluorescence of ZmRL6-GFP fusion protein was only observed in the nucleus, indicating ZmRL6 is located in the nucleus (Fig. 4a).

Yeast cells containing pGBKT7-ZmRL6 plasmid grew well on SD/-Trp/-His/-Ade medium and showed a positive reaction on SD/-Trp/-His/-Ade + X-α-gal medium, which are consistent with the positive control (Fig. 4b), thus suggesting ZmRL6 protein possesses transcriptional activity.

### ZmRL6 binding motifs revealed by DAP-seq

To investigate the regulatory mechanism mediated by *ZmRL6*, DAP-seq was performed and genes directly targeted by *ZmRL6* were uncovered. Using the Illumina platform (200-bp pair-end reads), ~ 9.7 and 10.9 million reads were produced from two biological repetitions. 81.98% and 81.25% of the ~ 9.7 and 10.9 million reads, respectively, have been uniquely mapped to the maize genome V4 (Table S4). Three typical ZmRL6 binding motifs (ACNGTT, NNACCNANN and AGCCNNAG) were identified (Fig. 4c). We predicted *ZmRL6*-binding sites by using the MACS2 software, setting the *P*-value < 0.05 (based on a Poisson distribution comparing the *ZmRL6* sample and the control) and uncovered 1535 peaks from two biological repetitions (Fig. 4d). We next analyzed the distribution of the peaks within these genes and found that 16.3% of them were located within the 5 kb upstream of the start codon, 14.2% were located in the 5 kb downstream of the stop codon, 2.93% were located in the exon regions and 1.95% were located in the intron regions (Fig. 4e).

In order to confirm the binding motifs of ZmRL6 (ACNGTT, NNACCNANN and AGCCNNAG), electrophoretic mobility shift assays (EMSAs) were performed subsequently with purified ZmRL6 proteins and a labelled DNA probe containing the ZmRL6-binding sites of ACCGTT, TTACCAAAC and AGCCCGAG. As shown in Fig. 4f, ZmRL6 was able to bind to ACCGTT, TTACCAAAC and AGCCCGAG. The addition of unlabeled competitors reduced the binding and the protein did not bind to the mutant probes. Without ZmRL6 proteins, only the band for the free probe was observed. These results confirmed that ZmRL6 can specifically bind to the motifs of ACCGTT, TTACCAAAC and AGCCCGAG.

### ZmRL6 target genes validation

**Fig. 5:**
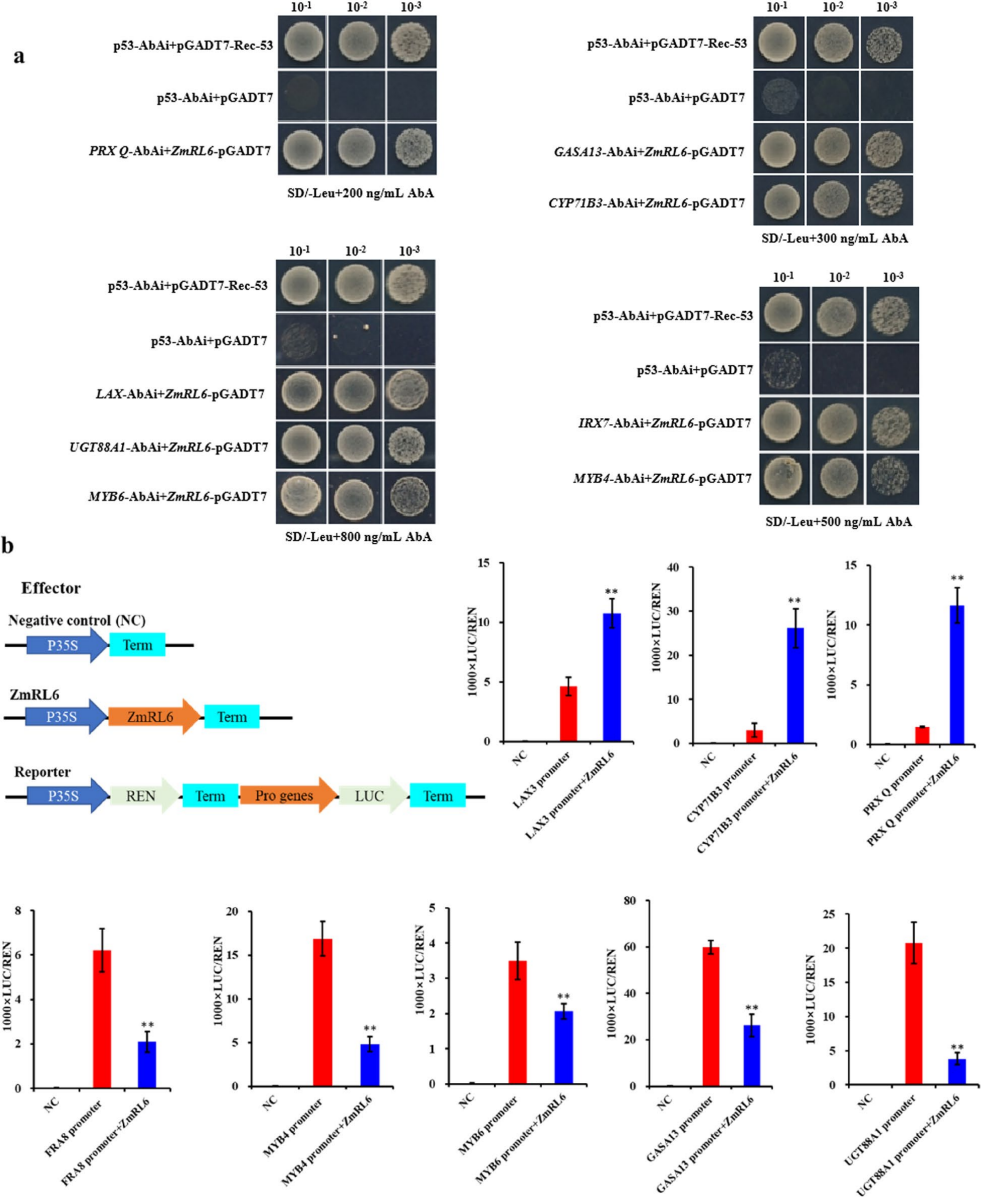
The Regulatory roles of ZmRL6 on its target genes. **a** Y1H assays showing the direct binding of ZmRL6 with its target genes (**b**) Schematic diagram of the dual luciferase system used in the study. The reporter constructs were transiently expressed in N. benthamiana leaves together with the control vector or 35S:ZmRL6 effector. The LUC/REN ratio represents the relative activity of the gene promoters. Data represent mean ± SD (*n* = 6) from three biological replicates. ** *p* < 0.01, Student’s *t* test relative to negative controls (NC)

By comparing overlapped genes between the RNA-seq and DAP-seq data, we were able to identify eight *ZmRL6* target genes, which were induced by drought stress (Fig. S5) These genes are mainly involved in phytohormone signal transduction (*Zm00001d042809*, *ZmLAX3*; *Zm00001d038056*, *ZmGASA13*), sugar metabolism (*Zm00001d043544*, *ZmUGT88A1*; *Zm00001d027311*, *ZmFRA8*), lignin synthesis (*Zm00001d012255*, *ZmMYB4*; *Zm00001d042665*, *ZmMYB6*) and antioxidative stress (*Zm00001d047495*, *ZmCYP71B3*; *Zm00001d037103*, *ZmPRXQ*) (Table S5). RNA-seq results showed that the expression levels of *ZmCYP71B3*, *ZmPRX Q* and *ZmLAX3* in the *ZmRL6*-OE plants were higher than that in the WT plants, and the expression levels of *ZmCYP71B3*, *ZmPRX Q* and *ZmLAX3* in *ZmRL6*-Mut plants were lower than that in the WT (Fig. S6). The expressions of *ZmGASA13*, *ZmMYB6*, *ZmMYB4*, *ZmIRX7* and *ZmUGT88A1* showed the opposite trend. RNA-seq assay showed *ZmRL6* inhibited the expression of *ZmGASA13*, *ZmUGT88A1*, *ZmIRX7*, *ZmMYB4* and *ZmMYB6* while promoting the expression of *ZmLAX3*, *ZmPRX Q* and *ZmCYP71B3*. 5The promoter sequence of those genes containing the conserved binding motifs (Table S6), further supported that they are the target genes of ZmRL6. To validate if ZmRL6 can directly bind to those promoters, we first conducted Y1H assays. As demonstrated in Fig. 5a, ZmRL6 can directly bind to the promoters of ZmPRX, ZmGASA13, ZmCYP71B3, ZmLAX3, ZmUGT88A1, ZmMYB6, ZmIRX7 and ZmMYB4. In contrast, the yeast cells co-expressing with empty vector did not show any growth (Fig. S7), indicating the specific binding of ZmRL6 with those target promoters. In addition, our dual luciferase assay showed that *ZmRL6* specifically represses LUC expression from the promoters of *ZmGASA13*, *ZmUGT88A1*, *ZmIRX7*, *ZmMYB4* and *ZmMYB6.* However, LUC expression increased from *ZmLAX3*, *ZmPRX Q* and *ZmCYP71B3 ZmMYB6* promoters; indicating that these genes are the target genes of *ZmRL6*, which is consistent with the RNA-seq results (Fig. 5b).

## Discussion

MYB TFs have been reported to play key roles in plant development and response to various abiotic and biotic stresses (Dubos et al., 2022). In our current study, we characterized the regulatory roles of *ZmRL6* in maize under drought stress. ZmRL6 was observed to localize to the nucleus and its ability for performing transcriptional activation was also confirmed. Moreover, *ZmRL6* was discovered to positively modulate the expression of several genes that are related to antioxidation, lignin synthesis, sugar metabolism, and phytohormone signaling pathways. Plant hormones play important role in response to abiotic stresses such as drought and high temperature. Under drought stress, the increase of ABA content can promote the stomatal closure, reduce the transpiration rate and water loss (Wang et al. 2018). Sucrose, acting as energy resource, participate in the mechanism of active oxygen scavenging systems and reduce oxidative damage (Ramel et al. 2009). The synthesis of lignin is closely related to phenylpropane, and phenols derived from phenylpropane pathway are important antioxidants, so phenylpropane biosynthesis pathway plays a crucial role in resisting various stress (Dixon, 1995). In transgenic Arabidopsis lines, over-expression of 3R-MYB gene *ZmMYB3R* was reported to confer drought tolerance (Wu et al. 2019).

Recent studies have revealed several key regulators that play essential roles in maize drought tolerance through affecting cell expansion, ROS production and ABA biosynthesis (Liu et al. 2020, 2021; Gao et al., 2022). To the best of our knowledge, we are the first to report the potential relevance of ZmRL6 in drought stress. To support this, we performed genetic transformation and provided lines of evidence including analyses of the phenotypes, physiological traits (RWC and EL) and antioxidant activities (MDA and proline levels) in maize. We found that the over-expression of *ZmRL6* in maize led to enhanced drought tolerance whereas *ZmRL6* deficient mutants led to attenuated drought tolerance. These results could be explained by the lower MDA contents detected in the *ZmRL6* transgenic plants as this implied the overexpression of *ZmRL6* could result in an improved tolerance to oxidative stress caused by drought stress. Since low MDAs and EL are important parameters that indicate membrane injury, we also examined these parameters and found that they were lower in the *ZmRL6*-overexpressing transgenic plants by comparing to those in the WT. These measurements indicated the degree of cell membrane damage in the transgenic plants caused by drought stress was less than that in the WT plants. However, an opposite trend of these results was found in the *ZmRL6* gene-knockout lines. Our results also suggested that ROS are scavenged by the enhanced activities of enzymatic antioxidants (SOD and POD) in response to drought stress. In addition, we found that sugar metabolism and oxidative stress-related biological processes were significantly enriched through GO annotation. Together, these data showed that *ZmRL6* may play a positive role in inhibiting cell membrane damage and cell death induced by drought. Our study uncovered ZmRL6 was a potential candidate gene that can regulate drought tolerance.

Romero reported that the classic motifs of MYB binding were: MBS I:TAACC/GGTT, MBSII:TAACTAAC, and MBSIIG: T/CACCA/TAC/AC (Romero et al. 1998). In our study, we identified the motifs of MYB classic bindings as MBS I: ACCGTT and MBS II G: TTACCAAAC, our DAP-seq further revealed that ZmRL6 also binds to the DNA sequence (cis-acting element) AGCCCGAG in the target genes to modulate transcription.

**Fig. 6:**
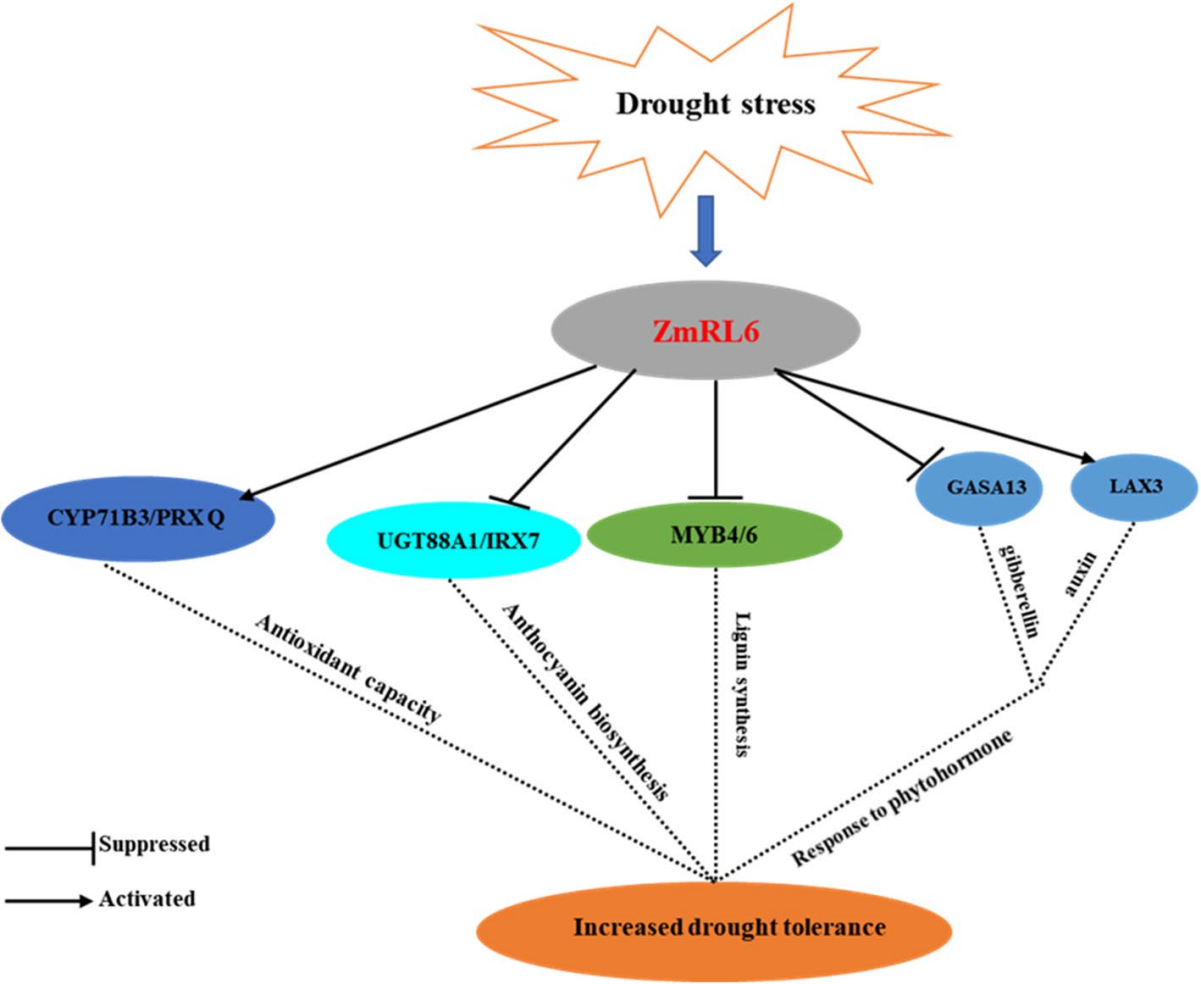
A schematic model of *ZmRL6 *in maize’s drought stress response regulatory network. ZmRL6 can activate and suppress CYP71B3/PRXQ: antioxidant enzyme genes, UGT88A1/IRX7: sugar metabolism genes, MYB4/6: lignin synthesis genes, and GASA13/LAX3: plant hormone genes to increase maize’s tolerance to drought

A series of *ZmRL6* target genes were identified by performing a combined analysis of the RNA-seq with DAP-seq data (Fig. S4). Eight of these *ZmRL6* potential target genes have been validated by dual luciferase assay. Taken all of these together, we have outlined a potential model for the molecular regulatory network of *ZmRL6* in drought stress response. We proposed one of the ways that *ZmRL6* may enhance drought tolerance in maize is through repressing the expression of phenylpropanoid metabolism related genes such as *ZmMYB4*, *ZmMYB6* and *ZmUGT88A1* (Fig. 6) because UGTs have been identified to involve in anthocyanin biosynthesis (Li et al. 2015; Zhao et al. 2007) and *At*MYB4 in Arabidopsis has been known to repress UV-induced sinapate ester biosynthesis, which derive from lignin intermediates (Zhao et al. 2007). Another means that *ZmRL6* may mediate drought stress is through phytohormones as they play a critical role in growth regulation under various biotic and abiotic stresses (Ha et al. 2013). In our study, ZmRL6 was revealed to possess activation and repression activities. In maize, ZmILI1 has been reported to have “dual” activities as it can repress the expression of Luc from the *ZmIAA26* promoter and also increase the expression from the *ZmLG1* promoter (Ren et al. 2020). We observed that *ZmRL6* inhibits the expression of a gibberellin-regulated related gene *ZmGASA13* and promotes the expression of an auxin transport related gene *ZmLAX3*. There are reports that indicate various abiotic stresses can reduce *ZmLAX* expression levels in roots (Yue et al. 2015) and over-expression of *AtGASA14*, a member of the Gibberellic Acid-Stimulated Arabidopsis (GASA) family, can suppress ROS accumulation (Sun et al. 2013). Therefore, *ZmRL6* may possibly enhance drought tolerance of maize by regulating auxin and gibberellin signaling pathways.

Based on the results from our study, it is also plausible that drought may trigger *ZmRL6* to induce signaling pathways that are associated with oxidative stress, lignin synthesis, sugar metabolism, and phytohormone signal transduction to mediate maize’s stress response. *ZmRL6* can regulate drought responsive genes in these signaling pathways through binding to the promoter of target genes (s) that have the motifs of ACCGTT, TTACCAAAC and AGCCCGAG. Drought stress is one of the predominant environmental factors that impedes plant growth and productivity. Our study has unfolded some of the potential mechanisms that maize can utilize to mediate drought. In particular, we have demonstrated the positive role of a 1R-MYB TF in maize’s drought stress response and ZmRL6 may serve as a candidate gene for designing and cultivating drought-tolerant maize to avoid severe yield loss in the future.

## Materials and methods

### Plant materials and stress treatment

Seeds of Yu882 were sown in a soil and vermiculite mixture (3:1) in a growth chamber at 25 ± 2◦ C under long-day conditions, with 16/8 h(light/dark) photoperiod cycles, 70% relative humidity, and a light density of approximately 300 μ mol m ^−2^ s ^−1^. When seedlings had three fully expanded leaves, they were divided into three groups. Plants of the second group were transferred into Hoagland nutrient solution (which was replaced every 2 days). The third group of plants was transferred into Hoagland’s nutrient solution for drought (20% PEG 6000). The second fully expanded leaves were sampled at 0, 12, 24, 48 60 h,72 h and 96 h, frozen in liquid nitrogen immediately and stored at -80 ◦C for qRT-PCR. Three plants from different treatments were used as biological replicates.

Maize seeds from different transgenic lines and the WT (Yu882) were sown in the flower pots (diameter: 15 cm; height: 15 cm) with homogeneous loam before allowing to grow in a greenhouse with the following conditions: temperature: 28℃, photon flux density: 700 μmol/m^2^/s, relative humidity: 50%-60% and light cycle: 16/8 h (light/dark). When the plants reached a 3-leaf stage, they were subjected to drought stress, which the plant’s soil water content was controlled at 20% for 20 days (The pot weight was measured twice a day to main the soil moisture at about 20% SWC) as described in (Chong et al. 2022). The controls had their soil’s water content controlled at about 80%. A total of 3 repetitions was performed for each group. For water loss measurements, the detached leaves from maize at the three-leaf seedling stage were exposed at room temperature. Leaves were weighed at various time intervals, and the loss of fresh weight (percentage of initial weight) was used to indicate water loss. At the same time, seedlings at the 3-fully expanded leaf stage were subjected to drought stress. In arid 10, 12, 13, 14 and 15 d, the number of surviving plants was counted. There were 20 plants per line and 3 replicates were set.

The RWC of the leaves was determined by using a drying-weighing method (Zygielbaum et al. 2009) whereas the REL was determined by an electrolyte exosmosis method (Zhang et al. 2010). The MDA content was determined by using the TBA method (Zhang et al., 2009). The proline content was measured by applying the ninhydrin colorimetric method (Guan et al. 2018). The SOD activity was assessed by the NBT reduction method, the POD activity was determined by the guaiacol colorimetric method (Pandey et al. 2016).

### RNA isolation and first strand cDNA synthesis

The total RNA from each sample was extracted using TRIzol reagent (Invitrogen, USA) per the manufacturer’s instructions. RNA integrity was checked by 1% agarose gel electrophoresis. First-strand cDNA was synthesized by PrimeScript RT reagent kit with gDNA eraser (TaKaRa Biotech, Kyoto, Japan).

### Gene cloning and sequence analysis of *ZmRL6*

The complete ORF of *ZmRL6* was amplified using the cDNA of Yu882 leaves in PCRs and constructed into a pMD19-T vector. The vector was then transformed into *Escherichia coli* DH5α cells and sequencing of the positive clone was performed by BGI Life Tech Co., Ltd. (Beijing). The primers are shown in Supplementary Table 1.

The amino acid sequences of the 1R-MYB proteins were analyzed by the Clustal X software. Protein domain and motif analyses of 1R-MYB are presented in the Pfam domains database (http://pfam.sanger.ac.uk). Phylogenetic tree was created by the neighbor-joining method with 1,000 bootstrap replicates using MEGA6.0 software. The predicted molecular weight and isoelectric point of ZmRL6 were analyzed by Expasy online software (https://web.expasy.org/protparam/).

### Quantitative real-time PCR (qRT-PCR) analysis

Gene-specific primers for qPCR were designed based on the corresponding sequences from Primer6 and are listed in Supplementary Table 1. Actin 18 s was used as an internal control. The qRT-PCR analyses were carried out by using SYBR Green PCR kit (TaKaRa) on the CFX96 Real Time System (Bio-Rad, Hercules, CA, USA) based on the manufacturer’s instructions. Three technical replicates were analyzed for each gene. Relative gene expression was calculated using the 2^−ΔΔCt^ method (Livak et al., 2001).

### Subcellular localization and transactivation activity assay

The coding sequence (CDS) of *ZmRL6* gene (excluding the stop codon) was amplified using PCR and the primers are shown in Supplementary Table 1. The cDNA sequence was cloned between the SpeI and AscI sites (underlined in primer sequences) of the pMDC83-GFP vector. The resulting 35S: ZmRL6-GFP and GFP control vector were transiently expressed in *Nicotiana benthamiana* leaves via Agrobacterium-mediated infiltration. mCherry serves as a positive nuclear marker. After two days, visualize the nuclei of the infected leaf tissues under a Zeiss LSM700 confocal microscope (Zeiss, Jena, Germany).

The *ZmRL6* gene was also transferred to pGBKT7 for an evaluation of the transactivation activity. The fusion plasmid pGBKT7-ZmRL6, pGBKT7-53/pGADT7-T (positive control) and pGBKT7-Lam/pGADT7-T (negative control) were transformed into *Saccharomyces cerevisiae* strain AH109 using the lithium acetate method. The yeast cells (50 μl) were spread onto SD/-Trp plates and incubated at 30℃ for 3–5 days. Individual yeast colony was cultured in YPDA liquid medium to an optical density of OD_600_ 0.8 at 30℃. After performing a 1:10, 1:100, 1:1000 and 1:10000 dilutions, the yeast cells were dropped onto SD/-Trp/-His/-Ade and SD/-Trp/-His/-Ade + X-α-gal plates to compare their ability to grow. The plates were next incubated at 30°C for 3–5 days before photographing.

### Vector construction for transgenic maize

The coding sequence of the *ZmRL6* gene was inserted into the *Asc*I and *BamH*I sites of the plasmid pFGC5941, which contained a constitutive ubiquitin promoter of maize. CRISPR-Cas9 was used to create mutations in the coding regions of *ZmRL6* to generate null alleles. sgRNAs were designed based on the B73 reference genome by the CRISPR-P 2.0 web-tool (https://www.genome.arizona.edu/crispr/CRISPRsearch.html) (Xing et al., 2014). sgRNA arrays were synthesized and cloned into a pGW-Cas9 construct. The resultant plasmid was then introduced into *A. tumefaciens* strain LBA4404 by utilizing the freeze–thaw method. Different transgenic plants were produced with *Agrobacterium*-mediated transformation (Ombori et al. 2013). PCR-positive transformed plants were then self-pollinated for two generations before detecting the transgene in the T_2_ plants using PCR. Primers for vector construction and transgenic plants identification are listed in Supplementary Table S1.

### RNA-seq and data analyses

The plump seeds of the *ZmRL6* over-expression mutant line (OE #10), knockout mutant line (Mut #14) and WT were selected and placed on a moist filter paper for incubation at 28℃. Germinated seeds were then planted in sandy soil and grown in the greenhouse at 28℃ with a photoperiod of 16/8 h (light/dark). When the maize seedlings grew to the three-leaf stage, the seedlings were subjected to drought stress for 10 days without watering, and the normally watered seedlings were taken as the control. Three plants from three different containers of each treatment were used as biological replicates. Leaves from OE #10, Mut #14 and WT under drought stress were sampled and immediately frozen in 80℃for RNA extraction. RNA sequencing and basic analysis were carried out at Genedenovo Biotechnology Co., Ltd (Guangzhou, China).

High-quality clean reads are attained by eliminating those reads containing adaptors, those reads containing more than 10% of unknown nucleotides (N), and those reads showing more than 50% of low-quality (Q value ≤ 20) bases. The clean reads were next mapped to the reference genome using Bowtie2 tool software (Langmead et al., 2012). The RNA-seq data were also mapped to the maize reference genome assembly B73_v4.0 using Hisat2 (version 2.0.5; https://ccb.jhu.edu/software/hisat2/index.shtml) aligner (Kim et al. 2015). Gene expression levels were estimated by FPKM (Florea et al. 2013). DEGs between the two sample groups were subsequently analyzed by using the DESeq R package. The |log_2_ (fold change) |≥ 1 and a false discovery rate (FDR) < 0.05 were set as the thresholds for determining DEGs with significance (Love et al. 2014). agriGO v2.0 (http://systemsbiology.cau.edu.cn/agriGOv2/index.php) was used to perform GO enrichment analysis (Tian et al., 2017). We have deposited our sequencing data to NCBI (PRJNA765933).

### DNA affinity purification sequencing (DAP-Seq) and data analysis

DAP-Seq experiments were performed by following the method as previously described (O'Malley et al. 2016). Briefly, a DAP-Seq genomic DNA (gDNA) library was prepared by attaching a short DNA sequencing adaptor onto a purified and fragmented gDNA. The adapter sequences were truncated to Illumina TruSeq adapters; the TruSeq Universal and Index adapters corresponded to the DAP-Seq Adapter A, CACGACGCTCTTCCGATCT, and Adapter B, GATCGGAAGAGCACACGTCTG. The DAP gDNA library was prepared using the kit from NEBNext® DNA Library Prep Master Mix Set for Illumina® (NEB no. E6040S/L). *ZmRL6* was fused to HaloTag using the kit from pFN19K HaloTag T7 SP6 Flexi Vector (cat. No. G184A) (Promega). *ZmRL6* fused to HaloTag was expressed using the TnT SP6 High-Yield Wheat Germ Protein Expression System (L3260) (Promega), before getting purified by using Magne HaloTag Beads (G7281) (Promega). Next, the *ZmRL6*-HaloTag mixture was incubated with 500 ng of DNA library in 40 µl phosphate-buffered saline (PBS) using slow rotation in a cold room for 1.5 h. The beads were washed five times with 200 μl PBS + NP40 (0.005%), and then resuspended into PBS. The supernatant was removed and 25 μl of EB buffer was added and incubated for 10 min at 98℃ to elute the bound DNA from the beads. The correct DAP-Seq library concentration for a specific read count was calculated based on the library fragment size. Negative control mock DAP-Seq libraries were prepared as described above but without the addition of protein to the beads.

We defined target genes as those that contain DAP-Seq peaks located within the transcribed regions of genes, in introns, 5 kb upstream of the TSS, or 5 kb downstream of the transcription termination site. DAP-Seq reads were aligned to the maize genome using Bowtie 2 (Langmead et al., 2012). DAP-Seq peaks were detected by MACS2 (Zhang et al. 2008). We used MACS version 2.0.10 with default parameters and duplicates allowance, with q-value < 0.05. Core motifs were identified by MEME-ChIP (Machanick et al.,^2011^).

### EMSA

The full-length *ZmRL6* cDNA was amplified with gene primers (Supplementary Table S1) and fused into the *Sgf*I and *Pme*I sites of pFN19K HaloTag® T7 SP6 Flexi® Vector. The HaloTag-ZmRL6 fusion protein was expressed using the TNT® Coupled Wheat Germ Extract Systems (Promega) and Magne® HaloTag Beads (Promega) EMSA. Oligonucleotide probes (Supplementary Table S1) were synthesized and labeled according to the standard protocol of Thermo Fisher Scientific (Shanghai, China). We used standard reaction mixtures for EMSA, which contained 20 ng of purified ZmRL6 fusion protein, 5 ng of biotin-labeled annealed oligonucleotides, 2 μl of 10 × binding buffer (100 mM Tris, 500 mM KCl, and 10 mM DTT, pH 7.5), 1 μl of 50% (v/v) glycerol, 1 μl of 100 mM MgCl_2_, 1 μl of 1 mg ml^–1^ poly (dI-dC), 1 μl of 1% (v/v) Nonidet P-40, and double-distilled water to obtain a final volume of 20 μl. The reactions were incubated at 25℃ for 20 min before electrophoresed in 6% (w/v) polyacrylamide gels and transferred to N + nylon membranes (Millipore, Darmstadt, Germany) in 0.53 × TBE (Tris–Borate-EDTA) buffer at 380 mA and 4℃ for 30 min. Biotin-labeled DNA was detected using the LightShift™ chemiluminescence EMSA kit (Thermo Fisher Scientific). Bands were subsequently visualized using the Chemiluminescent Western Blot Detection Kit (Thermo Fisher Scientific).

### Yeast one-hybrid assays

Yeast one-hybrid (Y1H) assays were performed according to the Matchmaker Y1H system manufacturer’s instructions (Clontech, Mountain View, CA, USA). Full-length ORFs of ZmRL6 were cloned into pGADT7 vector (Clontech); ~ 300 bp DNA fragments from indicated gene promoters were independently cloned into the pAbAi vector (Clontech) to generate reporter plasmids. The fusion pAbAi vectors were transformed into the Y1H Gold strain provided by Clontech and selected on synthetic dextrose (SD)/–Ura media. Then, the pGADT7-ZmRL6 vector was transformed into ‘Y1H Gold’ with the fusion pAbAi vector. ‘Y1H Gold’ containing the pGADT7-ZmRL6 vector was observed on selective SD plates without Leu plus AbA* (SD/–Leu/AbA*). Rec-p53 acted as a positive control, and p53 acted as negative controls. All of the primer information of the generated constructs is shown in Supplementary Table S1.

### Transient assays for in vivo reporter activation assays

The promoter region (~ 2500 bp) of ZmRL6 target genes were amplified and inserted into pGreenII0800-LUC. To generate the CaMV 35S promoter-driven *ZmRL6* effector, the full-length coding sequence of *ZmRL6* was inserted into pCAMBIA1300. Transient dual-luciferase assays were performed in *N. benthamiana* leaves and determined with dual-luciferase assay reagents (Promega Madison, WI, USA) as described in (Zhu et al. 2020; Guo et al. 2021). After infiltration for 48 h, LUC activity was measured using a GloMax®20/20 Luminometer (Promega, Cat# E5311). For this analysis, the ratio between LUC and REN activities was three times measured.

### Statistical analyses

Statistical analyses were carried out using SPSS 22.0 software. Data are presented as means and standard errors of the distributions. Student’s *t*-test or analysis of variance (ANOVA) were performed to determine the significance of differences between data sets. All tests were two-tailed, performed at the significance level α = 0.05. For all analyses, *p* < 0.05 was considered statistically significant (*p < 0.05; **p < 0.01).

### Supplementary Information

**Additonal file 1:** **Fig. S1.** The relative expression patterns of *ZmRL6* genes in response to drought stress by using qRT-PCR. **Fig. S2.** The survival rate of *ZmRL6* Transgenic lines under drought stress. **Fig. S3.** Quantitative determination of water loss of the detached leaves. **Fig. S4** Plant growth of the WT, OE and Mut lines of *ZmRL6*. **Fig. S5.** The relative expression patterns of eight target genes in response to drought stress by using qRT-PCR. **Fig. S6.** The expression level of eight target gene in RNA-seq. **Fig. S7.** The negative controls of Y1H assays showing no growth in the yeast selection medium.

**Additional file 2:** **Table S1.** Primer sequences used for experiments. **Table S2.** Data filtering statistics table of RNA-seq. **Table S3.** Comparison of reference statistics of RNA-seq. **Table S4.** Summary of reads analysis of DAP-seq. **Table S5.** The detailed information of eight target genes of *ZmRL6*. **Table S6.** Motif number of target gene.

## Data Availability

All data generated or analyzed during this study are included in this published article and/or on its supplementary information fles, as the following documents.

## Abbreviations

MYB: V-avian myeloblastosis viral oncogene homolog
ERF: Ethylene-responsive factor
bZIP: Basic leucine zipper
TFs: Transcription factors
ORF: Open reading frame
kDa: Kilodaltons
WT: Wild type
RWC: Relative water content
EL: Electrolyte leakage
MDA: Malondialdehyde
TBA: Thiobarbituric acid
NBT: Nitroblue tetrazolium
SOD: Superoxide dismutase
POD: Peroxidase
APX: Ascorbate peroxidase
CAT: Catalase
GO: Gene Ontology
CDs: Coding sequence
FPKM: Fragments per kilobase of transcript per million fragments mapped
FDR: False discovery rate
EMSA: Electrophoretic mobility shift assay
TSS: Transcription start site
AbA: Aureobasidin A

## References

[CR1] Baldoni E, Genga A, Cominelli E (2015). Plant MYB transcription factors: their role in drought response mechanisms. Int J Mol Sci.

[CR2] Cao LR, Lu XM, Zhang PY, Wang GR, Wei L, Wang TC (2019). Systematic analysis of differentially expressed maize ZmbZIP genes between drought and rewatering transcriptome reveals bZIP family members involved in abiotic stress responses. Int J Mol Sci.

[CR3] Cao YY, Zeng HX, Ku LX, Ren ZZ, Han Y, Su HH, Dou DD, Liu HF, Dong YH, Zhu FF, Li TY, Zhao QN, Chen YH (2020). ZmIBH1-1 regulates plant architecture in maize. J Exp Bot.

[CR4] Chen YH, Cao YY, Wang LJ, Li LM, Yang J, Zou MX (2018). Identification of MYB transcription factor genes and their expression during abiotic stresses in maize. Biol Plant.

[CR5] Chong L, Xu R, Huang PC, Guo PC, Zhu MK, Du H, Sun XL, Ku LX, Zhu JK, Zhu YF (2022). The tomato OST1-VOZ1 module regulates drought-mediated flowering. Plant Cell.

[CR6] Daryanto S, Wang LX, Jacinthe PA (2016). Global synthesis of drought effects on maize and wheat production. PLoS ONE.

[CR7] Dixon AR, Paiva NL (1995). Stress-induced phenylpropanoid metabolism. Plant Cell.

[CR8] Du H, Feng BR, Yang SS, Huang YB, Tang YX (2012). The R2R3-MYB transcription factor gene family in maize. PLoS ONE.

[CR9] Dubos C, Stracke R, Grotewold E, Weisshaar B, Martin C, Lepiniec L (2022). MYB transcription factors in Arabidopsis. Trends Plant Sci.

[CR10] Florea L, Song L, Salzberg SL (2013). Thousands of exon skipping events differentiate among splicing patterns in sixteen human tissues. F1000Research.

[CR11] Gao HJ, Cui JJ, Liu SX, Wang SH, Lian YY, Bai YT, Zhu TF, Wu HH, Wang YJ, Yang SP, Li XF, Zhuang JH, Chen LM, Gong ZZ, Qin F (2022). Natural variations of ZmSRO1d modulate the trade-off between drought resistance and yield by affecting ZmRBOHC-mediated stomatal ROS production in maize. Mol Plant.

[CR12] Guan C, Huang YH, Cui X, Liu SJ, Zhou YZ, Zhang YW (2018). Overexpression of gene encoding the key enzyme involved in proline-biosynthesis (PuP5CS) to improve salt tolerance in switchgrass (Panicum virgatum L.). Plant Cell Rep.

[CR13] Guo PC, Chong L, Wu FM, Hsu CC, Li CY, Zhu JK, Zhu YF (2021). Mediator tail module subunits MED16 and MED25 differentially regulate abscisic acid signaling in Arabidopsis. J Integr Plant Biol.

[CR14] Ha CV, Le DT, Nishiyama R, Watanabe Y, Sulieman S, Tran UT, Mochida K, Dong NV, Yamaguchi-Shinozaki K, Shinozaki K, Tran LP (2013). The auxin response factor transcription factor family in soybean: genome-wide identification and expression analyses during development and water stress. DNA Res.

[CR15] Kim D, Langmead B, Salzberg SL (2015). HISAT: A fast spliced aligner with low memory requirements. Nat Methods.

[CR16] Kobayashi F, Maeta E, Terashima A, Kawaura K, Ogihara Y, Takumi S (2008). Development of abiotic stress tolerance via bZIP-type transcription factor LIP19 in common wheat. J Exp Bot.

[CR17] Langmead B, Salzberg SL (2012). Fast gapped-read alignment with Bowtie 2. Nat Methods.

[CR18] Li YJ, Wang B, Dong RR, Hou BK (2015). AtUGT76C2, an Arabidopsis cytokinin glycosyltransferase is involved in drought stress adaptation. Plant Sci.

[CR19] Liu SX, Li CP, Wang HW, Wang SH, Yang SP, Liu XH, Yan JB, Li BL, Beatty M, Zastrow-Hayes G, Song SH, Qin F (2020). Mapping regulatory variants controlling gene expression in drought response and tolerance in maize. Genome Biol.

[CR20] Liu BX, Zhang B, Yang ZR, Liu Y, Yang SP, Shi YL, Jiang CF, Qin F (2021). Manipulating ZmEXPA4 expression ameliorates the drought-induced prolonged anthesis and silking interval in maize. Plant cell.

[CR21] Livak KJ, Schmittgen TD (2001) Analysis of relative gene expression data using real-time quantitative PCR and the 2(-DeltaDeltaC(T)) method, Methods 25402–408. 10.1006/meth.2001.1262 11846609

[CR22] Love MI, Huber W, Anders S (2014). Moderated estimation of fold change and dispersion for RNA-Seq data with DESeq2. Genome Biol.

[CR23] Machanick P, Bailey TL (2011). MEME-ChIP: motif analysis of large DNA datasets. Bioinformatics.

[CR24] O'Malley RC, Huang SC, Song L, Lewsey MG, Bartlett A, Nery JR, Galli M, Gallavotti A, Ecker JR (2016). Cistrome and epicistrome features shape the regulatory DNA landscape. Cell.

[CR25] Ombori O, Muoma JVO, Machuka J (2013). Agrobacterium-mediated genetic transformation of selected tropical inbred and hybrid maize (Zea mays L.) lines. Plant Cell Tissue Organ Culture.

[CR26] Pandey P, Srivastava RK, Rajpoot R, Rani A, Pandey AK, Dubey RS (2016). Water deficit and aluminum interactive effects on generation of reactive oxygen species and responses of antioxidative enzymes in the seedlings of two rice cultivars differing in stress tolerance. Environ Sci Pollution Res Intern.

[CR27] Paz-Ares J, Ghosal D, Wienand U, Peterson PA, Saedler H (1987). The regulatory c1 locus of Zea mays encodes a protein with homology to myb proto-oncogene products and with structural similarities to transcriptional activators. The EMBO J.

[CR28] Ramel F, Sulmon C, Bogard M, Couée I, Gouesbet G (2009). Differential patterns of reactive oxygen species and antioxidative mechanisms during atrazine injury and sucrose-induced tolerance in Arabidopsis thaliana plantlets. BMC Plant Biol.

[CR29] Ren ZZ, Wu LC, Ku LX, Wang HT, Zeng HX, Su HH, Wei L, Dou DD, Liu HF, Cao YY, Zhang DL, Han SB, Chen YH (2020). ZmILI1 regulates leaf angle by directly affecting liguleless1 expression in maize. Plant Biotechnol J.

[CR30] Romero I, Fuertes A, Benito MJ, Malpica JM, Leyva A, Paz-Ares J (1998). More than 80 R2R3 MYB regulatory genes in the genome of Arabidopsis thaliana. Plant J.

[CR31] Sun SL, Wang HX, Yu HM, Zhong CM, Zhang XX, Peng JZ, Wang XJ (2013). GASA14 regulates leaf expansion and abiotic stress resistance by modulating reactive oxygen species accumulation. J Experiment Botany.

[CR32] Sun XP, Xiang YL, Dou NN, Zhang H, Pei SR, Franco AV, Menon M, Monier B, Ferebee T, Liu T, Liu SY, Gao YC, Wang JB, Terzaghi W, Yan JB, Hearne S, Li L, Li F, Dai MQ (2022). The role of transposon inverted repeats in balancing drought tolerance and yield-related traits in maize. Nat Biotech.

[CR33] Tian T, Liu Y, Yan HY, You Q, Yi X, Du Z, Xu WY, Su Z (2017). AgriGO v2.0: a GO analysis toolkit for the agricultural community, 2017 update. Nucleic Acids Res.

[CR34] Wang YG, Fu FL, Yu HQ, Hu T, Zhang YY, Tao Y, Zhu JK, Zhao Y, Li WC (2018). Interaction network of core ABA signaling components in maize. Plant Molecular Biol.

[CR35] Wang GR, Yuan Z, Zhang PY, Liu ZX, Wang TC, Wei L (2020). Genome-wide analysis of NAC transcription factor family in maize under drought stress and rewatering. Physiol Molecular Biol Plants.

[CR36] Wu JD, Jiang YL, Liang YN, Chen L, Chen WJ, Cheng BJ (2019). Expression of the maize MYB transcription factor ZmMYB3R enhances drought and salt stress tolerance in transgenic plants. Plant Physiol Biochemist.

[CR37] Xing HL, Dong L, Wang ZP, Zhang HY, Han CY, Liu B, Wang XC, Chen QJ (2014). A CRISPR/Cas9 toolkit for multiplex genome editing in plants. BMC Plant Biol.

[CR38] Yue RQ, Tie SG, Sun T, Zhang L, Yang YJ, Qi JS, Yan SF, Han XH, Wang HZ, Shen CJ (2015). Genome-wide identification and expression profiling analysis of ZmPIN, ZmPILS, ZmLAX and ZmABCB auxin transporter gene families in maize (Zea mays L.) under various abiotic stresses. PLoS One.

[CR39] Zhang Y, Liu T, Meyer CA, Eeckhoute J, Johnson DS, Bernstein BE, Nusbaum C, Myers RM, Brown M, Li W, Liu XS (2008). Model-based analysis of ChIP-Seq (MACS). Genome Biol.

[CR40] Zhang L, Tian LH, Zhao JF, Song Y, Zhang CJ, Guo Y (2009). Identification of an apoplastic protein involved in the initial phase of salt stress response in rice root by two-dimensional electrophoresis. Plant Physiol.

[CR41] Zhang SJ, Li N, Feng G, Yang AF, Zhang JR (2010). Over-expression of TsCBF1 gene confers improved drought tolerance in transgenic maize. Mol Breeding.

[CR42] Zhang PY, Wang GR, Cao LR, Zhen Y, Ku LX, Wang TC, Wei L (2020). Analysis of Differentially expressed transcription factor genes in maize (Zea mays) under drought stress and re-watering. J Agricultural Biotechnol.

[CR43] Zhang PY, Qiu X, Fu JX, Wang GR, Wei L, Wang TC (2021). Systematic analysis of differentially expressed ZmMYB genes related to drought stress in maize. Physiol Mol Biol Plants.

[CR44] Zhao JF, Zhang WH, Zhao Y, Gong XM, Guo L, Zhu GL, Wang XC, Gong ZZ, Schumaker KS, Guo Y (2007). SAD2, an importin β-like protein, is required for UV-B response in Arabidopsis by mediating MYB4 nuclear trafficking. Plant Cell.

[CR45] Zhu YF, Huang PC, Guo PC, Chong L, Yu GB, Sun XL, Hu T, Li Y, Hsu CC, Tang K, Zhou Y, Zhao CZ, Gao W, Tao WA, Mengiste T, Zhu JK (2020). CDK8 is associated with RAP2.6 and SnRK2.6 and positively modulates abscisic acid signaling and drought response in Arabidopsis. New Phytol.

[CR46] Zygielbaum AI, Gitelson AA, Arkebauer TJ, Rundquist DC (2009). Non-destructive detection of water stress and estimation of relative water content in maize. Geophys Res Lett.

