# Supplementary material for: Molecular mechanism analysis of ZmRL6 positively regulating drought stress tolerance in maize
Source: Stress Biol. 2023 Nov 16;3(1):47. doi: 10.1007/s44154-023-00125-x (PMC10654321; doi:10.1007/s44154-023-00125-x)
Supplement: Supplementary file 1 — Additonal file 1: Fig. S1. The relative expression patterns of ZmRL6 genes in response to drought stress by using qRT-PCR. Fig. S2. The survival rate of ZmRL6 Transgenic lines under drought stress. Fig. S3. Quantitative determination of water loss of the detached leaves. Fig. S4 Plant growth of the WT, OE and Mut lines of ZmRL6. Fig. S5. The relative expression patterns of eight target genes in response to drought stress by using qRT-PCR. Fig. S6. The expression level of eight target gene in RNA-seq. Fig. S7. The negative controls of Y1H assays showing no growth in the yeast selection medium. [file 44154_2023_125_MOESM1_ESM.docx]

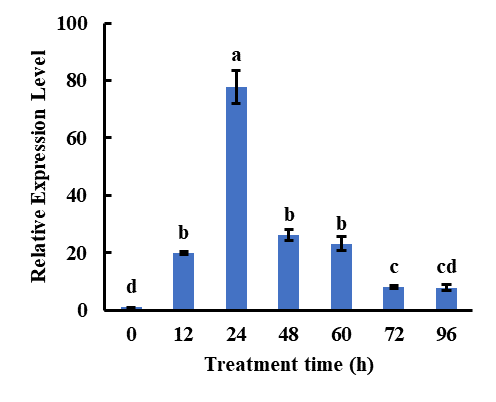


**Fig.S1** The relative expression patterns of *ZmRL6* genes in response to drought stress by using qRT-PCR. Data represent the mean value ± standard deviation (SD) (n = 3). Different letters indicate significant differences at 0.05 level.


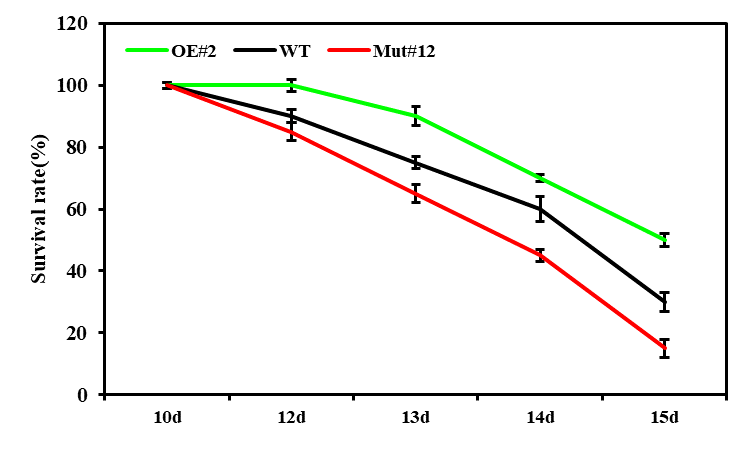


**Fig. S2** The survival rate of *ZmRL6* Transgenic lines under drought stress.


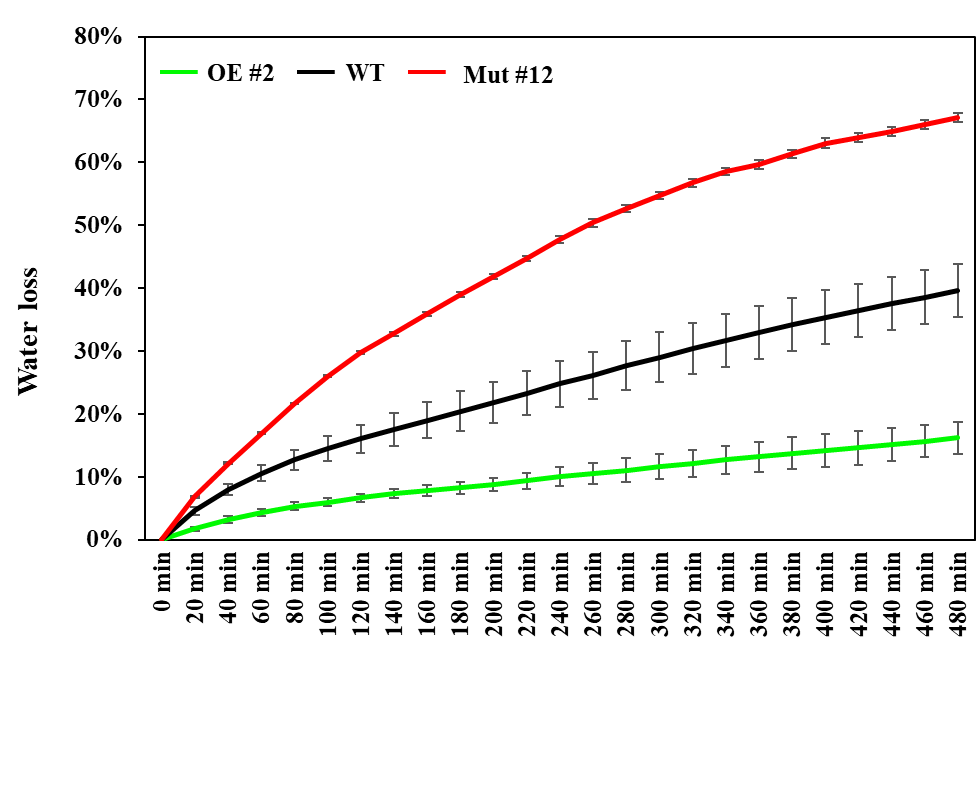


**Fig. S3** Quantitative determination of water loss of the detached leaves. Values are mean ± SE from three independent experiments. Three different leaves at similar stages were used for each experiment.


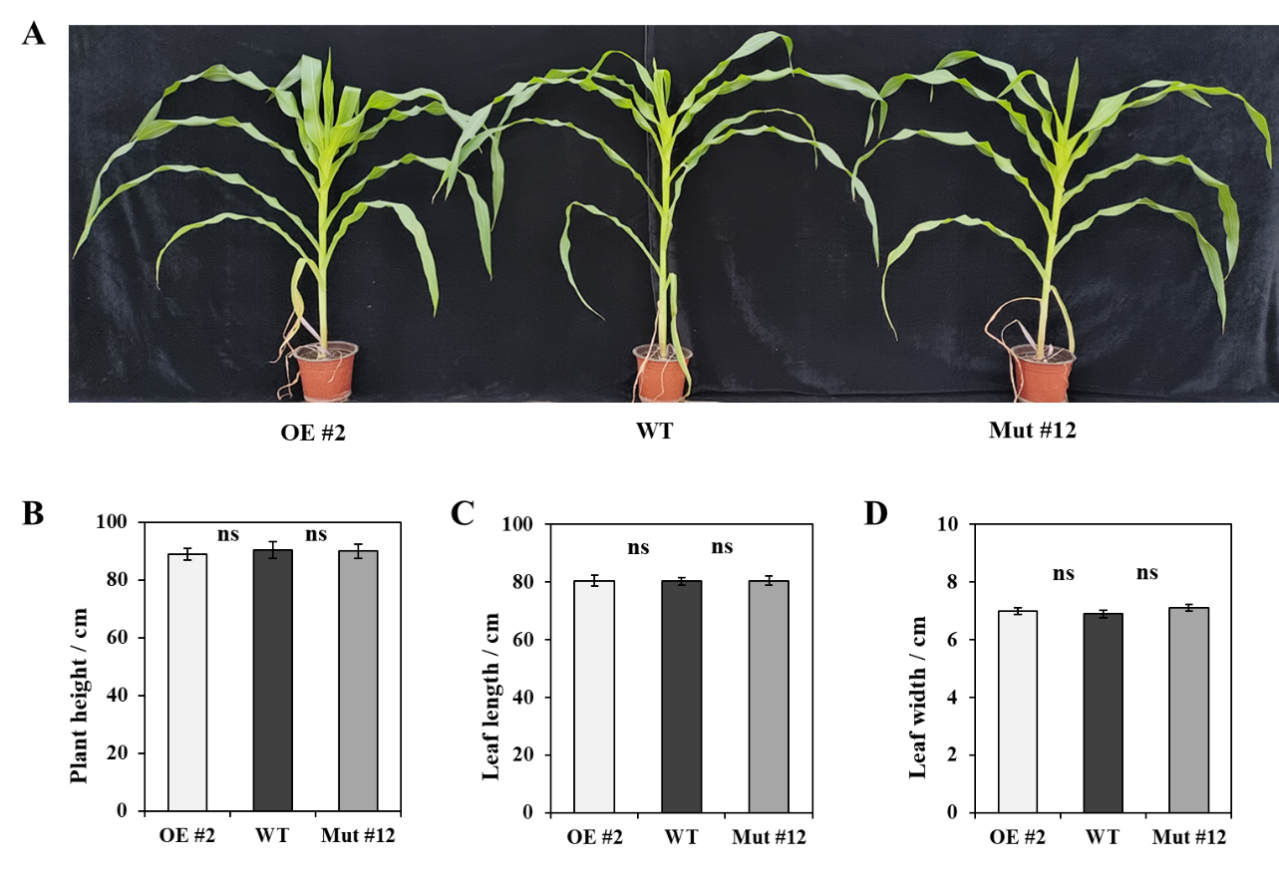


**Fig. S4** Plant growth of the WT, OE and Mut lines of *ZmRL6*. (**A**) Plant growth morphology of WT, OE and Mut lines. Phenotype of plant height (**B**), leaf length (**C**) and leaf width (**D**) in WT, OE and Mut lines (n = 10). Data are shown as the mean ± SE and the P value is estimated by the one-way ANOVA analysis. ns: not significant. Scale bar = 10 cm in (**A**).


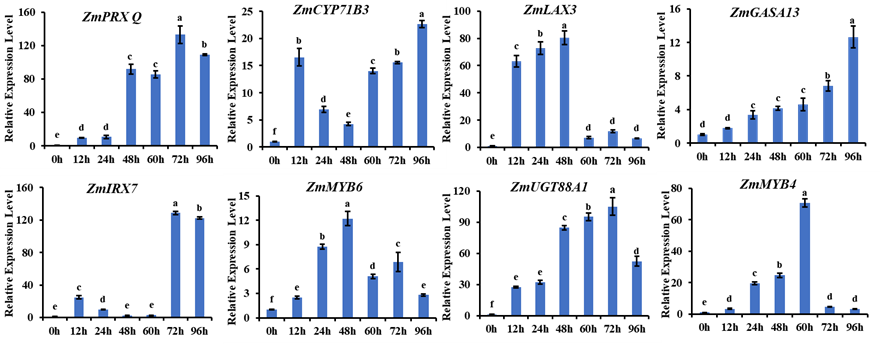


**Fig. S5** The relative expression patterns of eight target genes in response to drought stress by using qRT-PCR. Data represent the mean value ± standard deviation (SD) (n = 3). Different letters indicate significant differences at 0.05 level.


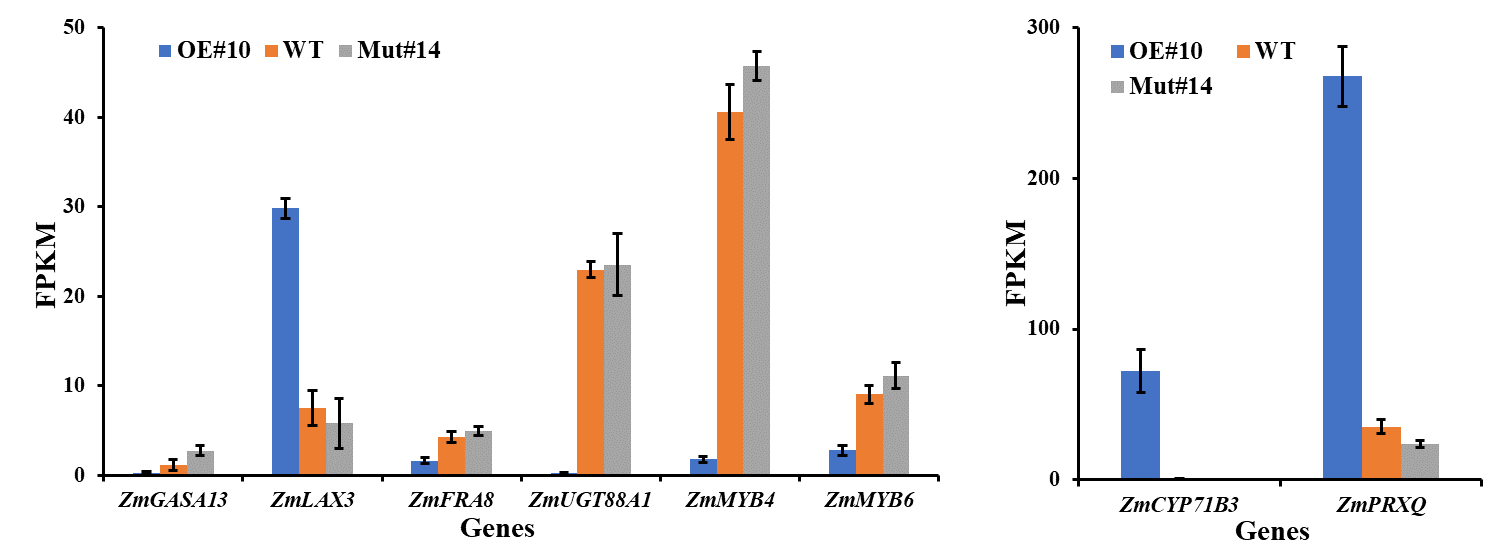


**Fig. S6**  The expression level of eight target gene in RNA-seq


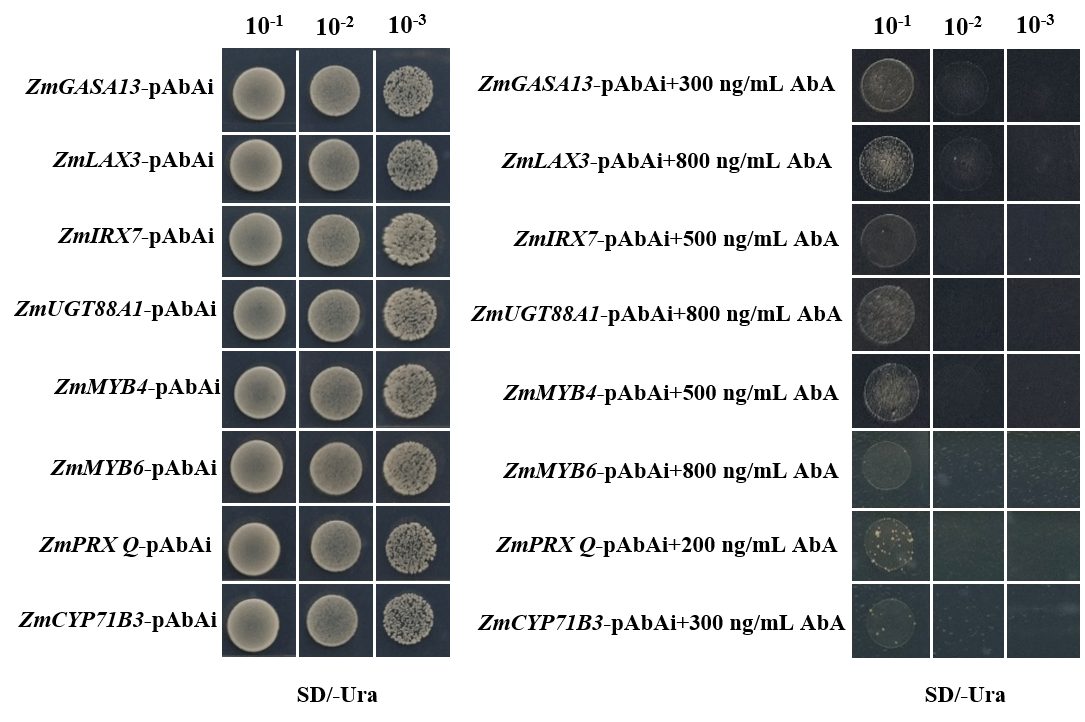


**Fig. S7** The negative controls of Y1H assays showing no growth in the yeast selection medium.
