# Supplementary material for: Molecular mechanism analysis of ZmRL6 positively regulating drought stress tolerance in maize
Source: Stress Biol. 2023 Nov 16;3(1):47. doi: 10.1007/s44154-023-00125-x (PMC10654321; doi:10.1007/s44154-023-00125-x)
Supplement: Supplementary file 2 — Additional file 2: Table S1. Primer sequences used for experiments. Table S2. Data filtering statistics table of RNA-seq. Table S3. Comparison of reference statistics of RNA-seq. Table S4. Summary of reads analysis of DAP-seq. Table S5. The detailed information of eight target genes of ZmRL6. Table S6. Motif number of target gene. [file 44154_2023_125_MOESM2_ESM.docx]

**Table S1**. Primer sequences used for experiments

| Gene | Forward Primer (F) | Reverse Primer （R) |  |
| --- | --- | --- | --- |
| FL-ZmRL6 | AAGAAGCAGCAGACAGGA | GAGGGAGAGGGAACAGAA | cloning premiers of full-length cDNA |
| qRT-ZmRL6 | CGGTGTACGACAAGGACAC | CCTCCAGCAGCTCGTAGTA | qRT-PCR |
| 18S | CCTGCGGCTTAATTGACTC | GTTAGCAGGCTGAGGTCTCG |  |
| *ZmPRX Q* | CACCCTCAAGGACCAGAACG | CGGCGGGGTAGAAGTAAACC |  |
| *ZmGASA13* | CATTCCCGCCCCCAATGT | TTCTTGTTGCCGTAGGTGCC |  |
| *ZmCYP71B3* | AACATCCCGGACTTGTTCCC | GCCCATGATCTTTTCGTCGC |  |
| *ZmLAX3* | ACCACGTCATCCAGTGGTTC | GCAGGAACGTGCAGTTGAAG |  |
| *ZmIRX7* | TGTGATCCCACCTCATGTGC | GCGATTGCGGCCATAATGTT |  |
| *ZmMYB4* | CGGCTTCGCTGGATGAACTA | CGATGAGCGACCACTTGTTG |  |
| *ZmUGT88A1* | ACGTCTCGCTGAATAGCCTG | TCAGCAGTCCTTGGCCATTT |  |
| *ZmMYB6* | CTGGATGAACTACCTCCGCC | TTGATCTCGTTGTCCGTCCG |  |
| OE-ZmRL6 | TT GGCGCGCC ATGGCGTCGCTCTCGATG | CG GGATCC CTAGATCTTCAGCTGTTT | OE-vector |
| Mut-ZmRL6 | AATAATGGTCTCAGGCG GCGCTGGCGGTGTACGACA | ATTATTGGTCTCTAAAC GCGGGGTAGCGGTAGGCGG | Mut-vector |
|  | G GCGCTGGCGGTGTACGACA GTTTTAGAGCTAGAAATAGC | GCGGGGTAGCGGTAGGCGG CGCTTCTTGGTGCC |  |
|  |  |  |  |
|  |  |  |  |
| GFP-ZmRL6 | GG ACTAGT ATGGCGTCGATCTAGATG | TT GGCGCGCC AGATCTTCAGCTGTTTCA | Subcellular localization |
| KT-ZmRL6 | CG GAATTC ATGGCGTCGATCTAGATG | CGC GGATCC CTAGATCTTCAGCTGTTT | transcriptional activation assay in yeast |
| 19K-ZmRL6 | AGCGATAAGGCGATCGCC ATGGCGTCGATCTAGATG | GCCCGAATTCGTTTAAAC CTAGATCTTCAGCTGTTT | protein expression |
| AACGGT | TGC**AACGGT**CTTCACCAACCCAACGGTCCA | TGGACCGTTGGGTTGGTGAAGACCGTTGCA | Probe for EMSA |
| TTACCAAAC | CG**TTACCAAAC**TACCTAATTCCTTACCAAAC | GTTTGGTAAGGAATTAGGTAGTTTGGTAACG |  |
| CCACCTACC | AAT**CCACCTACC**GATCCACCTACCTCCACCTACC | GGTAGGTGGAGGTAGGTGGATCGGTAGGTGGATT |  |
| AGCCCGAG | TTCGACAGCCCGAGAATCAG | CTGATTCTCGGGCTGTCGAA |  |
| ZmGASA13 | TAGGGCGAATTGGGTACC GTGGTGAACCAACTAGAT | GAGGGGGGGCCCGGTACC AGTATGACTAGGGAATGGTA | transient assays for in vivo activation activity |
| ZmLAX3 | TAGGGCGAATTGGGTACC GCTCCCCTTCAGATGCAT | GAGGGGGGGCCCGGTACC TTTATAACAAGCGCCTTGCA |  |
| ZmFRA8 | TAGGGCGAATTGGGTACC GAGGGGTTGTGTGGTTTT | GAGGGGGGGCCCGGTACC TTGCTGAGCGTTCCAAGTCT |  |
| ZmUGT88A1 | TAGGGCGAATTGGGTACC GTCGAGTCTGTCTTCCAT | GAGGGGGGGCCCGGTACC CCTGCGGGACCCAACTTTAT |  |
| ZmMYB4 | TAGGGCGAATTGGGTACC CTGTTGCTGTACAAATGC | GAGGGGGGGCCCGGTACC TTGCCTTGCGCTTGCTTGAT |  |
| ZmMYB6 | TAGGGCGAATTGGGTACC TGACTTCTCAGCCAAGGT | GAGGGGGGGCCCGGTACC GATGAAGCGTGGATTTTATT |  |
| ZmPRXQ | TAGGGCGAATTGGGTACC TCAAAGCAAGAGACACCA | GAGGGGGGGCCCGGTACC AAACATCTTGCGATGGTTGG |  |
| ZmCYP71B3 | TAGGGCGAATTGGGTACC CGAGTAGATTGCCCCATC | GAGGGGGGGCCCGGTACC TTCGTGAGTTCAATCACTTG |  |
| ZmPRX Q | GATGAATTGAAAAGCTTATTCACCAAGGCA | ACAGATCCCCGGGTACCGGTCGACGAACAATAT | Y1H |
| ZmGASA13 | GATGAATTGAAAAGCTTGTATGGTGGTGCGT | ACAGATCCCCGGGTACCAACGCTTGGTTCAGC |  |
| ZmCYP71B3 | GATGAATTGAAAAGCTTCCTATTGATTGACAG | ACAGATCCCCGGGTACCCACTACATAAATTGC |  |
| ZmLAX3 | GATGAATTGAAAAGCTTCTAGCTCCGCTTCTT | ACAGATCCCCGGGTACCGAATGTGGCGCTTGC |  |
| ZmIRX7 | GATGAATTGAAAAGCTTACTCAACTAACTAGG | ACAGATCCCCGGGTACCGGTGTGCCAACGGGC |  |
| ZmMYB4 | GATGAATTGAAAAGCTTGATCTTGACTTAATT | ACAGATCCCCGGGTACCGATATACTCGCGGAC |  |
| ZmUGT88A1 | GATGAATTGAAAAGCTTTCGGTGCGGCTA | ACAGATCCCCGGGTACCAGGGGTACCCTCAAG |  |
| ZmMYB6 | GATGAATTGAAAAGCTTGTCTCCGCTAGCGGG | ACAGATCCCCGGGTACCCAACTTTTTAGATAC |  |

Table S2 Data filtering statistics table of RNA-seq

| **Sample** | **RawDatas** | **CleanData(%)** | **Adapter(%)** | **LowQuality(%)** | **polyA(%)** | **N(%)** |
| --- | --- | --- | --- | --- | --- | --- |
| Mut-1 | 37120458 | 36629932 (98.68%) | 37956 (0.10%) | 451694 (1.22%) | 0 (0.00%) | 876 (0.00%) |
| Mut-2 | 47511068 | 46881866 (98.68%) | 46962 (0.10%) | 581450 (1.22%) | 0 (0.00%) | 790 (0.00%) |
| Mut-3 | 45089278 | 44529486 (98.76%) | 43370 (0.10%) | 515622 (1.14%) | 0 (0.00%) | 800 (0.00%) |
| OE-1 | 49498910 | 48818996 (98.63%) | 45860 (0.09%) | 632778 (1.28%) | 0 (0.00%) | 1276 (0.00%) |
| OE-2 | 39614014 | 39089422 (98.68%) | 39940 (0.10%) | 483736 (1.22%) | 0 (0.00%) | 916 (0.00%) |
| OE-3 | 37460510 | 36938536 (98.61%) | 31858 (0.09%) | 489226 (1.31%) | 0 (0.00%) | 890 (0.00%) |
| WT-1 | 39358508 | 38775248 (98.52%) | 48382 (0.12%) | 533884 (1.36%) | 0 (0.00%) | 994 (0.00%) |
| WT-2 | 50054824 | 49383894 (98.66%) | 52616 (0.11%) | 617072 (1.23%) | 0 (0.00%) | 1242 (0.00%) |
| WT-3 | 45177206 | 44524848 (98.56%) | 47632 (0.11%) | 603588 (1.34%) | 0 (0.00%) | 1138 (0.00%) |

**Table S3** Comparison of reference statistics of RNA-seq

| Sample | Total | Unmapped(%) | Unique_Mapped(%) | Multiple_Mapped(%) | Total_Mapped(%) |
| --- | --- | --- | --- | --- | --- |
| Mut-1 | 36429502 | 3055884 (8.39%) | 32268204 (88.58%) | 1105414 (3.03%) | 33373618 (91.61%) |
| Mut-2 | 46666734 | 4068364 (8.72%) | 41166147 (88.21%) | 1432223 (3.07%) | 42598370 (91.28%) |
| Mut-3 | 44251694 | 3793948 (8.57%) | 39065524 (88.28%) | 1392222 (3.15%) | 40457746 (91.43%) |
| OE-1 | 48481278 | 4081553 (8.42%) | 42751594 (88.18%) | 1648131 (3.40%) | 44399725 (91.58%) |
| OE-2 | 38895548 | 3240815 (8.33%) | 34207569 (87.95%) | 1447164 (3.72%) | 35654733 (91.67%) |
| OE-3 | 36718006 | 3174382 (8.65%) | 32256974 (87.85%) | 1286650 (3.50%) | 33543624 (91.35%) |
| WT-1 | 38434206 | 3341437 (8.69%) | 33889831 (88.18%) | 1202938 (3.13%) | 35092769 (91.31%) |
| WT-2 | 48939682 | 4015116 (8.20%) | 43366379 (88.61%) | 1558187 (3.18%) | 44924566 (91.80%) |
| WT-3 | 44243670 | 3808786 (8.61%) | 39089434 (88.35%) | 1345450 (3.04%) | 40434884 (91.39%) |

**Table S4** Summary of reads analysis of DAP-seq

| Sample | Raw reads | clean reads | Ratio (%) |
| --- | --- | --- | --- |
| ZmRL6-Ⅰ | 1183607 | 970322 | 81.98 |
| ZmRL6-Ⅱ | 1348830 | 1095924 | 81.25 |
| Input-Ⅰ | 3629565 | 2876391 | 79.24 |
| Input-Ⅱ | 3707234 | 3027465 | 81.66 |

**Table S5** The detailed information of eight target genes of *ZmRL6*

| Gene ID in maize | Chr | Upstream location of TSSs | Gene name in maize | Function description | Gene ID in Arabidopsis | Gene name in Arabidopsis |
| --- | --- | --- | --- | --- | --- | --- |
| *ZmGASA13* | 3 | 2782 | ZmGASA13 | Auxin transporter-like protein 1 | AT3G10185.1 | GASA13 |
| *ZmLAX3* | 6 | 2963 | ZmAUX1 | Gibberellin-regulated protein 13 | AT2G38120.1 | AUX1 |
| *ZmFRA8* | 3 | 1720 | ZmIRX7 | UDP-glycosyltransferase 88A1 | AT2G28110.1 | IRX7 |
| *ZmUGT88A1* | 1 | 2934 | ZmUGT88A1 | Probable glucuronoxylan glucuronosyltransferase IRX7 | AT3G16520.1 | UGT88A1 |
| *ZmMYB4* | 8 | 4909 | ZmMYB4 | MYB DNA-binding domain superfamily protein | AT4G38620.1 | MYB4 |
| *ZmMYB6* | 3 | 499 | ZmMYB6 | Transcription repressor MYB6 | AT4G09460.1 | MYB6 |
| *ZmPRXQ* | 9 | 3436 | ZmPRX Q | Premnaspirodiene oxygenase | AT3G26060.1 | ATPRX Q |
| *ZmCYP71B3* | 4 | 1898 | ZmCYP71B3 | methyltransferase PMT15 | AT3G26220.1 | CYP71B3 |

**Table S6** Motif number of target gene

| gene ID | gene name | Motif number | | |
| --- | --- | --- | --- | --- |
|  |  | AACGGT | TTACCAAAC | AGCCCGAG |
| Zm00001d037103 | *ZmPRX Q* | 1 | 1 | 0 |
| Zm00001d038056 | *ZmGASA13* | 1 | 0 | 0 |
| Zm00001d047495 | *ZmCYP71B3* | 1 | 0 | 0 |
| Zm00001d042809 | *ZmLAX3* | 0 | 0 | 1 |
| Zm00001d027311 | *ZmIRX7* | 3 | 0 | 0 |
| Zm00001d012255 | *ZmMYB4* | 1 | 0 | 0 |
| Zm00001d043544 | *ZmUGT88A1* | 0 | 0 | 1 |
| Zm00001d042665 | *ZmMYB6* | 1 | 0 | 0 |
